# Influencing factors for high quality care on postpartum haemorrhage in the Netherlands: patient and professional perspectives

**DOI:** 10.1186/s12884-015-0707-9

**Published:** 2015-10-23

**Authors:** Mallory D. Woiski, Evelien Belfroid, Janine Liefers, Richard P. Grol, Hubertina C. Scheepers, Rosella P. Hermens

**Affiliations:** Department of Obstetrics and Gynaecology, Radboud University Medical Centre, Nijmegen, P. O. Box 9101, 6500 HB Nijmegen, The Netherlands; Scientific Institute for Quality of Hospital and Integrated Care (IQ healthcare), Radboud University Medical Centre, P.O. Box 9101, 6500 HB Nijmegen, The Netherlands; Department of Obstetrics and Gynaecology, Maastricht University Medical Centre, P.O. Box 5800, 6202 AZ Maastricht, The Netherlands

**Keywords:** Postpartum haemorrhage, Pregnancy, Quality indicators, Guideline adherence, Barriers, Facilitators, Implementation

## Abstract

**Background:**

Postpartum haemorrhage (PPH) remains a major contributor to maternal morbidity even in high resource settings, despite the development and dissemination of evidence-based guidelines and Advance-Trauma-Life-Support (ATLS) based courses for optimal management of PPH. We aimed to assess current influencing factors (obstacles and facilitators) for the delivery of high quality PPH-care from both patient and professional perspective.

**Methods:**

We qualitatively explored influencing factors for delivering high quality PPH-care, by having individual interviews with PPH-patients and focus group interviews with the different types of professionals working in the delivery room. For both perspectives, the theoretical frameworks of Grol and Cabana were used to classify the influencing factors for optimal PPH-care (factors of the guidelines, of professionals, of patients, of the social setting and of the organisation). In order to assess the importance of the influencing factors found among the professionals, we quantified these factors in a web-based questionnaire.

**Results:**

A total of 12 patients and 41 professionals participated in the interviews, and 315 complete surveys were analyzed. The main obstacle for high quality PPH-care identified by patients was the lack of information given by the professionals to the patient and partner before, during and after the PPH event. An informative patient website, a patient leaflet and a follow-up consultation were mentioned as facilitators. The main obstacles according to the professionals were: lack of clarity of the guidelines, lack of knowledge and failing team-communication. Team training and checklists/ flowcharts were considered facilitators.

**Conclusions:**

Different obstacles to the delivery of high quality PPH-care were identified by both patients and professionals. These data can be used to develop a focused strategy to improve PPH-care.

**Trial registration:**

NCT 00928863

## Background

Postpartum haemorrhage (PPH) remains to be the leading cause of severe maternal morbidity in several high-income countries [1–3]. Moreover, PPH rates continue to increase in these countries, including the Netherlands where the incidence of PPH increased from 3 % in 2003 to 7 % in 2011 [4–6]. It is common knowledge that enhanced adherence to evidence-based guidelines and better technical and non-technical skills improve patient care and outcome [7–9]. However, the development and dissemination of evidence-based PPH guidelines (intended to assist professionals and patients in the prevention and management of PPH-care) or the introduction of training innovations such as Advance Trauma Life Support (ATLS) based courses (to improve knowledge and technical and non-technical skills among teams dealing with obstetric emergencies such as PPH) are not enough to close the existing gap between guidelines, course-instructions and daily practice [10–12]. Substandard care is regularly mentioned for women with a PPH [1, 13, 14]. In a French study, in 38 % of the women with a PPH of more than 1500 ml and in 70 % of the women who died as a result of a PPH, suboptimal care factors were detected .

In fact, PPH-care consists of two phases, the prevention and the treatment phase, where professionals give routine care followed by emergency care. Different action must be taken by different professionals, consecutively or simultaneously, in a limited timeframe [15, 16]. Streamlining PPH-care for every professional, founded on evidence-based PPH guidelines and ATLS-based course instructions, is necessary to provide high quality PPH-care [17]. Proper implementation of these guidelines and instructions is therefore essential and can only be achieved once the causes for not following guidelines and course instructions on different levels have been identified and overcome [12, 18]. Therefore, to improve PPH-care, an in-depth analysis identifying influencing factors (both obstacles and facilitators) for the delivery of high quality PPH-care will provide information for focussing an implementation strategy to improve this care [19]. Currently, little is known about contemporary obstacles and facilitators for high quality PPH-care from both patient and professional perspective. Therefore, the objective of this study is to perform an in-depth analysis to identify obstacles and facilitators for providing high quality PPH-care, from both patient and professional perspective. This knowledge will make it possible to develop a focussed implementation strategy to improve PPH-care.

## Method

### Setting

To explore and classify the influencing factors for delivering high quality PPH-care from both patient and professional perspective, two theoretical frameworks were used, the frameworks of Grol and Cabana [20, 21] in particular. These frameworks facilitate exploration and description of potential barriers using five categories: guideline factors and recommendations (I); factors of the professionals who should use the guideline (II); factors of patients who have to accept or contribute to using the guideline (III); social setting factors (e.g., colleagues of the involved professionals) (IV); and organisational factors (V) [22–25]. The Committee on research Involving Human Subjects of the region Arnhem-Nijmegen of the Netherlands assessed the study and concluded that our study (ABR no. NL25975. 091.08) would be carried out in accordance with the applicable rules concerning the review of research ethics committees and informed consent.

### Design and population

#### Patients

To explore the influencing factors for high quality PPH-care from the patient perspective, a qualitative study among patients with postpartum haemorrhage in the past was performed through semi structured one-on-one interviews. Patients who delivered a baby and lost more than 1000 ml of blood after delivery were eligible for inclusion. Patients were asked to participate by means of a notice on childbirth forums on the Internet in order to obtain as many variations as possible in hospitals throughout the country (www.babybytes.nl, www.zwangerschapspagina.nl). PPH-patients who delivered in two different university hospitals were also approached by letter to contact us if they were willing to participate. We excluded patients who had a delivery in primary care or had a Caesarean Section because we mainly wanted to evaluate the care in delivery rooms and not in operating rooms.

#### Professionals

To explore the influencing factors for high quality PPH-care from the professional perspective, four focus group interviews were conducted with four different groups of professionals involved in the Dutch PPH-care: 1. obstetricians, 2. obstetricians in training, 3. midwives working under the supervision of an obstetrician and 4. obstetric (OB) nurses working in the delivery rooms. Professionals from 21 different hospitals [University Hospitals (UH), Teaching Hospitals (TH) and Non-Teaching Hospitals (NTH) with a similar distribution by type across the country] were invited to participate. In all the participating hospitals we contacted the obstetrician in charge of the obstetric division by email and requested one or two delegates per type of professional aforementioned to discuss obstacles and facilitators in their daily postpartum haemorrhage care. Information concerning the dates and place of the meetings was included in the request and if we did not get a reply of attendance within 2 weeks, we telephoned the obstetrician as a reminder.

#### Surveys among the professionals

In order to quantify the identified influencing factors (obstacles and facilitators) from the focus group interviews of professionals, so as to assess the importance of the influencing factors using the same theoretical frameworks, a national questionnaire survey was held among the four different professional groups. For this survey, all Dutch obstetricians and obstetricians in training (*n* = 1230) received the questionnaire through the e-mail service of the Dutch Society of Obstetrics and Gynaecologists (NVOG). Additionally, contact information of midwives was retrieved from a national registration of midwives working in secondary care. They were all sent an invitation letter with the link to the questionnaire (*n* = 175). As no national registration existed for OB-nurses, we approached the head-nurses of delivery rooms of 26 Dutch hospitals to distribute the web link of the questionnaire to their personnel. Since we did not directly contact the OB-nurses, the number of approached OB-nurses is unknown. In order to get as much response as possible, all professionals got a reminder.

### Data collection

#### Patients

Patients were informed about the study and informed consent and permission to audiotape the semi-structured interview was obtained. The one-on-one interviews took 30 to 45 min and were conducted individually by two experienced researchers (MW, EB). The semi-structured interviews gave the patients the chance to talk freely, as well as to express their personal feelings about the experienced obstacles and facilitators for optimal care. Interviews were structured in the following manner: we asked them to describe their experience with PPH-care received in all phases of the care procedure (during pregnancy at the outpatient clinic, during and after delivery and in the follow-up phase of the outpatient clinic). As soon as obstacles or facilitators came up we explored them in detail, using the two theoretical frameworks (guideline-, professional-, patient-, social setting- and organisational factors). Data collection was finalized when no new influencing factors were found and saturation was reached [26].

#### Professionals

A chairperson with expertise in PPH-care moderated the focus group interviews*.* All participants were informed about the study and informed consent and permission to audiotape the interview was obtained. The structure of the interview was based on previously developed quality indicators and the two theoretical frameworks. The quality indicators, which were based on PPH-guidelines and ATLS-based course instructions, consisted of the following five domains: 1) Prevention of PPH, 2) Management of patients with >500 ml blood loss, 3) Management of patients with >1000 ml blood loss or with signs of shock and 4) Organisation of PPH-care and 5) Management of patients with >2000 ml blood loss [27]. All participants were asked to mention obstacles and facilitators for providing high quality care on the subjects of the first four domains, particularly regarding adherence to evidence-based guidelines and ATLS-based course instructions. In addition, the obstetricians were asked about influencing factors for optimal care in the fifth domain (patients >2000 ml blood loss); midwives and nurses did not take care of patients with >2000 ml blood loss.

The focus group interviews were structured in the following manner: we asked respondents to describe obstacles and facilitators regarding the specific quality indicators. We explored more specifically whether in their own hospital they experienced any obstacle or facilitator of the five categories of the theoretical frameworks: guideline, professional, patient, social setting and organisational factors.

#### Surveys among the professionals

For the national survey, the influencing factors found in the four focus group interviews were converted into a web-based questionnaire using Limesurvey (https://manual.limesurvey.org). The questionnaire consisted of two sections: first, general information such as age, gender and profession; and second, 103 Likert-scale items regarding the identified obstacles and facilitators from the focus group interviews. The 103 items were structured in categories according to the same theoretical frameworks we used in previous projects [22, 25]. If necessary, questions were transformed so that the answers ‘agree’ or ‘totally agree’ displayed an obstacle (5 point Likert-scale, ranging from totally agree to totally disagree). The questions were adjusted to the different professional groups based on the content of their work. There was room for comment at the end of the questionnaire. The questionnaire did not accept unanswered items; however, it was possible to stop doing the questionnaire at any time. In those cases, the completed questionnaires were not saved and only the attempt to do so was registered. The questionnaire was tested by an epidemiologist and gynaecologist (RH, HS) and adjusted if a question was not clear enough.

### Analysis

#### Patients and professionals

The interviews were fully transcribed and the obstacles and facilitators were extracted separately by two researchers (EB, MW) with the qualitative program Atlas.ti (version 6.2.23, Atlas.ti Scientific Software Development GmbH; Berlin, Germany), and categorised according to the two theoretical frameworks. The transcripts and categorisation were (re)-read by MW, EB, HS, and RH to ensure reliability of the data. Differences in coding were discussed and final decisions on items and categories were made in consensus.

#### Surveys among the professionals

The questionnaire data were gathered in an electronic database and analysed descriptively in terms of frequencies using IBM SPSS Statistics (Version 20.0. Armonk, NY: IBM Corp). The percentages of responders who considered an item an obstacle were calculated on all 103 items by combining the score ‘totally agree’ and ‘agree’ of the 5 point Likert-scale. We analysed the obstacles, both for the whole group of professionals and for the four different groups of professionals. To assess the reliability of the questionnaire, internal consistency per domain was calculated by Cronbach’s alpha.

## Results

### Study population

#### Patients

Twelve patients participated in the semi-structured interviews. In the 11th interview no new information was acquired, nor in the 12th interview, meaning saturation was reached. Three patients derived from the two university hospitals and 9 from the forum. Five patients had a one-on-one interview at the request of the patient, 7 were interviewed by telephone. The patients delivered in 11 different hospitals. The median blood loss post-partum was 3.4 litres and the median age 28.5 years. All types of hospitals (university hospitals, non-university-, teaching and non-teaching hospitals) were represented.

#### Professionals and survey among professionals

In total, 41 professionals participated in the four focus group interviews, of which nine obstetricians (from 8 different hospitals), eight obstetricians in training (from 6 different hospitals), fifteen midwives (from 10 different hospitals) and nine OB-nurses (from 9 different hospitals). Seventeen percent of the professionals worked in a non-teaching hospital, 46 % in a teaching hospital and 36 % in a university hospital. The four different types of professionals and the different types of hospitals in combination with the distribution of hospitals across the country display a diverse group of professionals and different care settings.

The survey with questionnaires yielded 499 responses of which 318 were complete. Three were excluded because the questionnaire was not completed by a target group member. In total, 315 questionnaires were used for analysis; 37 % concerned obstetricians, 30 % obstetricians in training, 19 % midwives and 14 % OB-nurses. Table 1 outlines the general information of the respondents. These respondents include all types of obstetrical caregivers working in Dutch delivery rooms. The Chronbach’s alpha for the questionnaire was more than 0.820 and that renders the questionnaire reliable.

**Table 1:** General characteristics of professionals from the completed surveys (quantitative study)

		N (318)	(%)
Gender	Male	64	20
	Female	254	80
Position	Obstetricians	119	37
	Obstetricians in training	94	30
	Midwifes	61	19
	Nurses	44	14
Type of hospital	University Hospital	105	33
	Teaching Hospital	155	49
	Non Teaching Hospital	58	18
N^o^. of deliveries per year	<1000	38	12
	1001–1500	110	35
	1501–2000	75	24
	>2000	95	30

### Influencing factors from patient perspective

**Fig. 1:**
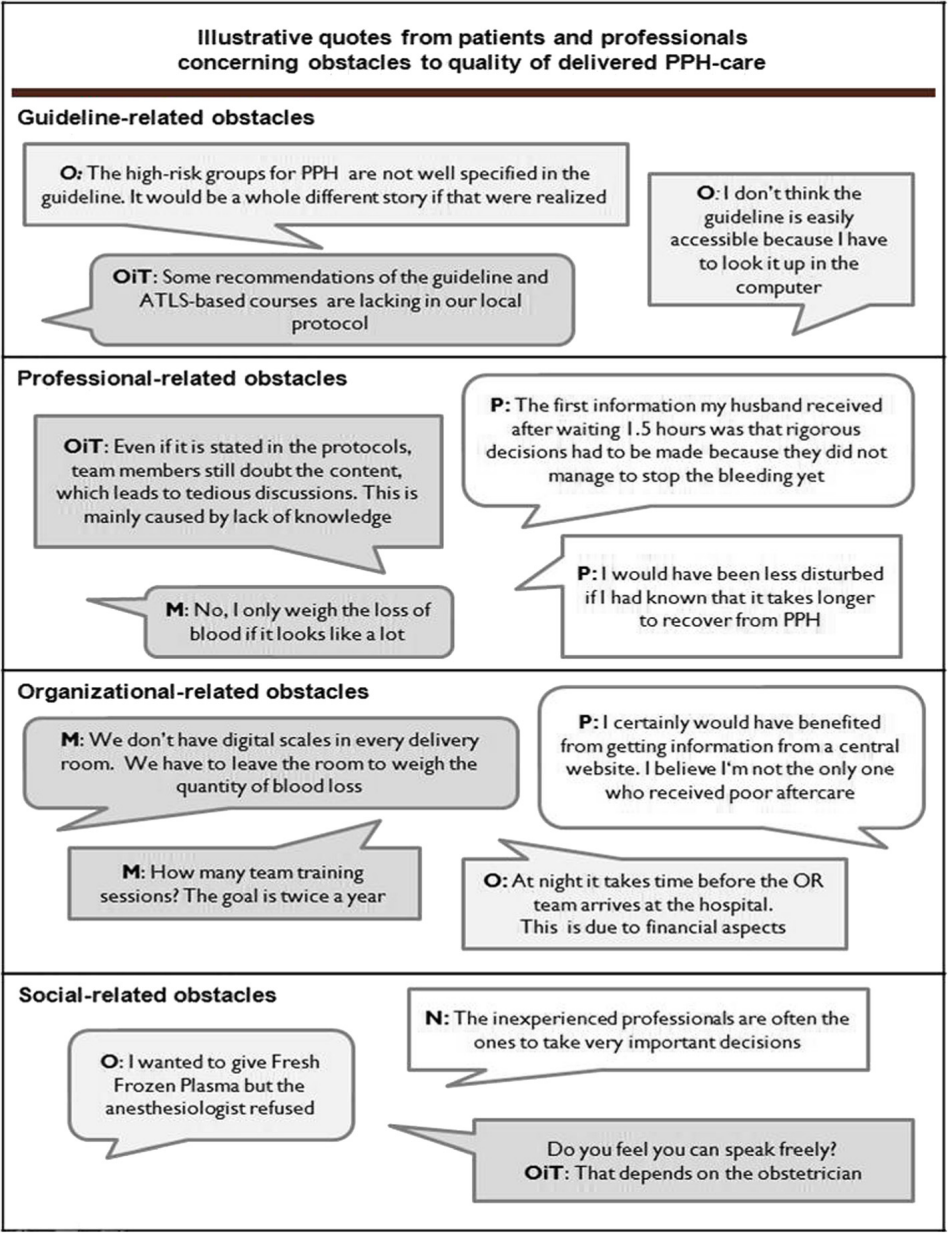
Illustrative quotes from patients and professionals concerning obstacles to quality of delivered PPH-care

From the patient interviews, we identified 38 obstacles and 4 facilitators in the five domains of the two theoretical frameworks (domain of the guideline, professional, patient, social setting and organisation). Most obstacles were cited at professional and organisational levels. The main influencing factors for high quality PPH-care per domain are shown in Table 2 and described beneath. Figure 1 illustrates quotes, inter alia, from PPH-patients.

**Table 2:** Obstacles and facilitators related to guideline and ATLS-based course adherence according to patients (qualitative-study)

Domain	Obstacles	Stated by N^o^ of patients (*n* = 12)
The professionals (*n* = 18)	Poor information to the patient about PPH	9
	Poor information to the partner/family about the patient’s medical condition, the risks and medical procedures	7
	Patient feels not being taken seriously by the professional	6
	Professionals panic when PPH occur	4
	Incorrect/no information about policy of future deliveries	4
The organisation (*n* = 7)	Lack of information material like folders and website	7
	The patient has to deal with many different clinicians	3
Facilitator (*n* = 4)	Patient information material/website is facilitating for patient information	3

#### Professional factors

Patients particularly mentioned the poor information provision about PPH (9 patients). They often received no information or incorrect information on the risk factors for PPH and the medical procedures, and had no knowledge prior to delivery of their risk for PPH. If PPH occurred, patient, partners and family were not informed or received insufficient information on the medical condition of the patient. They received insufficient or confusing information on the risks and medical procedures during the treatment of PPH, and they thought that the professionals showed panic when PPH occurred. In the recovery period, patients received inconsistent information on the duration of recovery and the policy of future deliveries. Moreover, patients often felt not being taken seriously by health care professionals.

#### Organisational factors

Patients noted that they did not receive an informative leaflet (7 patients) and that extensive digital information was not available. Patients identified the need for receiving information about PPH during pregnancy, an informative patient website, a patient leaflet about PPH and a follow-up talk as facilitators. In addition, premature discharge from the hospital and dealing with many different clinicians were considered obstacles by at least three patients.

### Influencing factors from professional perspective (interviews and survey)

In total, 83 obstacles and 30 different facilitators for high quality PPH-care were identified in the four focus group interviews. Obstetricians identified 43 unique obstacles, obstetricians in training 39, midwives 47 and the OB-nurses 31. We selected the most frequently cited influencing factors that were identified in at least 3 out of 4 focus group interviews (Table 3), categorised according to our frameworks: 1. The guideline; 2. Professional; 3. Patient; 4. Social setting; and 5. Organisation.

**Table 3:** Obstacles and facilitators related to guideline and ATLS-based course adherence according to professionals (focus group interview results: qualitative study)

Domain (N^o^ barriers found)	Obstacles	Mentioned in N^o^ of interviews
Guideline (*n* = 18)	Items of the PPH-guideline and ATLS-based course instructions are not included in the local hospital protocol	4
	The PPH-guideline is difficult to obtain at the delivery ward	3
	Recommendations and definitions in the PPH-guideline are unclear	3
Professional (*n* = 28)	Professionals lack awareness regarding the importance of the recommendations of the guideline and ATLS-based course	4
	Professionals experience a feeling of time pressure	4
	Professionals overestimate their knowledge regarding identifying the patient-categories at risk for PPH and regarding the treatment of high-risk patients and patients with PPH	4
	Professionals are overconfident regarding their ability to estimate the blood loss without the use of a weighing-scale	4
	Professionals lack to detect high-risk patients at the outpatient clinic	4
Social setting (*n* = 7)	Lack of communication in the team responsible for the patient, about the risks, policy, seriousness of the situation or actions that need to be taken	4
	Uncertain leadership caused by lack of knowledge about each other’s knowledge and expertise. This is caused by inexperienced professionals and frequent change of team composition	4
	Disagreement between team members and with personnel of other disciplines about the seriousness of the situation (blood-bank personnel and anaesthesiologists)	3
	Lack of team collaboration as orders are not followed and team members prefer following their own instincts in treatments, which leads to inconsequent policy	3
	Presence of hierarchy leads to dread, for team members find it difficult to call in a gynaecologist who is at home and speak freely against the supervisor when there is a disagreement about policy	3
Organisation (*n* = 30)	Materials necessary for treatment of patients with PPH are not direct available	3
	Shortage of (qualified) staff	3
	Skills/team trainings are not organised or not organised on a regular basis	3
	Lack of practical tools at the delivery rooms, such as checklist/flowchart for easier and practical use of the guideline	3
	Lack of finance	3
	Complication discussions are not organised on a structural basis because it is too time consuming	3
Facilitators (*n* = 30)	The availability of a checklist/flowchart about PPH at the delivery rooms would improve care	4
	Training on using a checklist/flowchart about PPH would improve care	4
	Skills/team trainings on a regular basis improve care	3

For the quantification by means of questionnaires, we used all influencing factors found. Table 4 shows the main influencing factors for the different types of professionals, and in total, identified in the survey. Presented are the influencing factors with total scores of 25 % or more. We will discuss these factors per category in more detail, using more in-depth information of the focus group interviews as well. Figure 1 represents quotes of the professionals regarding the different domains.

**Table 4:** Obstacles according to professionals (web-based survey results: quantitative study)

Domain: Guideline	Overall %	Obstetricians %	Obstetricians in training %	MidWifes %	Nurses %
The national guideline lacks a flowchart to use in acute situations	55	54	69	48	39
My local protocol does not say you should establish a policy for the delivery of a high-risk patient^a^	39	33	38	48	43
My local protocol does not say you should consider a manual placenta removal at 500 ml blood loss^a^	39	34	36	59	30
I have to find out myself that there is an update of the guideline	35	29	36	34	50
The guideline is difficult to obtain in our delivery room	27	27	23	30	34
My local protocol does not say you should weigh blood loss for every high-risk patient^a^	26	17	27	27	25
You cannot use the national PPH-guideline in acute situations	25	24	29	25	16
Domain: Professional					
Measuring the urine output is low on my list of priorities	57	44	65	66	55
I don’t have enough skills to perform surgical interventions (B-lynch etc.)	50	30	77	NA	NA
Professionals are not aware that warm saline infusion is beneficial	50	35	63	61	50
The recommendations for >1000 ml blood loss are less important when a patient lost 1000 instead of 1500 ml	25	23	30	31	14
We do not weigh the blood loss for every high-risk patient when it is estimated as little	36	24	49	44	32
I don’t have enough knowledge to perform surgical interventions (B-lynch etc.)	27	7	53	NA	NA
I don’t have enough knowledge about bimanual compression	26	11	33	56	NA
Domain : Social setting					
Lack of experience of the team members with the use of warm saline infusion	50	45	53	56	48
Working with inexperienced obstetricians (in training) is an obstacle	30	20	36	34	39
Domain: Organisation					
There is a need for more skills and drills	53	42	67	57	50
In my hospital it is not possible to give a patient warm saline infusion	50	40	49	59	68
Complication discussions are not multidisciplinary	44	31	65	43	34
Time is an obstacle for organising skills and drills	38	36	51	31	27
Not every delivery room has material to measure urine output	36	18	33	57	61
The multidisciplinary arrangements are not tight enough	33	2	42	30	27
Organising debriefings is too time consuming	32	29	39	33	23
Complication discussions are not organised on a regular basis	30	17	48	31	25
Facilitators					
A flowchart about PPH in the delivery room would improve care	63	50	73	68	58
A checklist about PPH in the delivery room would improve care	57	51	56	63	60
There is a need for more skills and drills	53	42	67	57	50
A second gynaecologist on duty for only emergencies would help me to quickly consult an extra gynaecologist	30	26	34	NA	NA

*NA* questions not applicable for these professionals

^a^Respondents without a local protocol were excluded from this question (*n* = 12)

#### Guideline factors

Tables 3 and 4 show the results of the most important factors related to the guideline. The most frequently cited factor (55 %) was the need for a flowchart or checklist in the delivery room, particularly among the obstetricians in training (69 %). Another important factor cited was the lack of inclusion of main guideline recommendations or ATLS-based course instructions in the local protocols. The most frequently cited missing items were ‘to establish a policy for the delivery of a high-risk patient’ (39 %) and ‘manual placenta removal at 500 ml blood loss’ (39 %). Other important missing items were: weighing the blood loss for every high-risk patient (26 %) and recommendations regarding the prevention of PPH (20 %). The missing items were particularly important for the midwives (highest scores). Other obstacles in this domain were related to the availability of the guideline/local protocol in the delivery rooms (25 %). This was particularly important for the OB-nurses (34 %).

#### Professional factors

In the domain of the professionals we found factors related to attitude, knowledge and skills (Tables 3 and 4). Professionals lacked awareness about the importance of some recommendations causing that these recommendations were skipped or had a lower priority to be enforced, such as measuring the urine output (57 %) and weighing the blood loss for every high-risk patient (36 %), among obstetricians in training and midwives in particular. In all focus group interviews, professionals mentioned that they could properly estimate the amount of blood loss without using a weighing-scale. Also an overestimation of proper knowledge was cited, for example, of which patient-category is at risk. Professionals’ knowledge related to the benefits of warm saline infusion in PPH (50 %), surgical interventions (27 %) (both highest for obstetricians in training) and bimanual compression (26 %), particularly among midwives, could be improved. Skills related to surgical interventions (50 %) fall short, particularly among obstetricians in training (77 %). In all focus group interviews time pressure was considered another major reason not to follow the guideline, but this did not score higher than 25 % in the survey.

#### Patient factors

Professionals did not mention influencing factors at the patient level.

#### Social setting factors

In the social setting (Tables 3 and 4), for all professionals, in general the main obstacles were related to working with inexperienced physicians in training (30 %). An obstacle cited in all focus group interviews, but without scores over 25 % in the survey, was a lack of communication about the policy on the delivery of the high-risk patient, the steps to be taken and the steps already taken.

Besides communication, team collaboration (following orders from team members) and hierarchy (criticising the actions of a leading team member) were cited in all focus group interviews, in particular the lack of clarity in leadership, uncertainty about knowledge and experience levels of team members, resulting in a lack of confidence regarding their skills and ability. The frequent changes in staff and working in different team compositions with inexperienced professionals were considered causes for these problems. Professionals indicated that skills- and team training (53 %) were important facilitators.

#### Organisational factors

In the domain of the organisation (Tables 3 and 4), professionals and in particular obstetricians in training (67 %) mentioned a need for more frequent skills- and team training (53 %). In addition, professionals stated that skills- and team training was not organised at all (11 %) or not multidisciplinary (12 %) (data not shown). Time (38 %) and cost (14 %) were obstacles for organising these training sessions. Another main obstacle was the lack of material available for providing warm saline infusion (50 %). Material to measure urine output (36 %) and for high-pressure fluid replacement (19 %), and monitoring facilities (16 %) were lacking in the delivery rooms as well.

In addition, according to the respondents, discussions on complications were often not performed multidisciplinary (44 %) and not organised on a regular basis (30 %) because this was considered time-consuming (32 %). Moreover, multidisciplinary arrangements lacked clarity and concreteness (33 %).

Professionals indicated that flowcharts/checklists (63 %/57 %) in the delivery rooms could be important facilitators for the delivery of high quality PPH-care. Obstetricians in training, midwives and OB-nurses in particular thought the use of checklists/flowcharts could be helpful, because multiple actions had to be performed in a very short period of time. The use of these tools should be incorporated in skills-and team training, leading to a proper application.

## Discussion

This study is the first to describe an in-depth analysis to identify influencing factors (obstacles and facilitators) for providing high quality PPH-care, from both patient and professional perspective. The main obstacle from the patients’ perspective was at a professional level; predominantly the lack of information provided by the professionals to the patient, partner or family, before, during and after the PPH event. An informative patient website regarding PPH and a follow-up consultation were mentioned as facilitators.

The obstacles identified by the professionals were in all domains, except the patient domain. Their main obstacles were: lack of clarity of the guideline, absence of various guideline recommendations in local protocols, lack of knowledge and failing team communication. Team training and checklists or flowcharts were considered facilitators for better care.

The lack of communication and information provision to patients and family is a frequent obstacle found not only in this study, but also in studies in other areas of healthcare [24, 28, 29]. As regards PPH this was also observed in a simulated setting, where not any team member addressed the family members to let them know what was going on with their loved one during the PPH simulation [30]. It may be that informing the patient and family, especially in an emergency situation, is not the first thing that is done. However, above all in an emergency situation, the patient and family are vulnerable and scared, and diagnostic uncertainty or lack of information will leave a negative impression [31]. Although PPH can suddenly emerge, care providers can nonetheless anticipate on risk factors, especially if a high risk for PPH is present, by giving the patient information beforehand, during pregnancy, about the risks. Patients and family can seek information about PPH and be a partner in their own care. A study by Harrison et al. regarding patient satisfaction in high risk pregnancies, reported that the majority of the women wanted to be an active partner in their own care [32]. In other areas of healthcare, an active patient participation has led to better outcomes [33, 34]. As the patients mentioned in our study, an active patient participation can be supported by the development of a reliable, informative website, and a patient leaflet about PPH. Moreover, in this study, professionals did not mention obstacles at the patient level, which means that this factor requires extra attention.

A common obstacle from professional perspective in literature is the poor quality of the guidelines and protocols [21, 22, 29]. Particularly the lack of clarity and concreteness of the guideline for application in normal practice and the lack of essential recommendations from both guidelines and course instructions in the local PPH-protocols were mentioned in this study. For PPH-care, these are main obstacles, since PPH-care is characterised by two phases: The prevention phase (performing routine care); followed by the treatment phase (emergency-care phase) where different action must be taken by different professionals, consecutively or simultaneously, in a limited timeframe [15, 16]. Streamlining PPH-care, according to clear, descriptive protocols that are founded on concrete evidence-based guidelines and ATLS-based course instructions, is necessary for every professional to provide high quality care [12, 35]. However, guideline recommendations are rarely specified in precise behavioural terms such as who does what, when, where, and how, and therefore local protocols are essential to close the gap between best evidence and practice [36–38]. Proper implementation of evidence-based PPH-guidelines and ATLS-based courses are essential for high quality PPH-care and can only be achieved once the causes for not following guidelines and instructions on different levels have been identified and overcome [7, 39]. From literature it is known, that transformation of guideline recommendations into clear and descriptive local protocols requires time, skills in protocol development and convincing evidence or guideline recommendations [40, 41]. Furthermore, different studies report lack of agreement with guideline recommendations by the professionals [42, 43]. The use of checklists and flowcharts, based on evidence-based guidelines and ATLS-based course instructions, could be important facilitators for the delivery of high quality PPH-care, particularly in case of performing multiple actions in a limited timeframe. Use of checklists and flowcharts has been proven effective in critical care [44–46]. This is indeed indicated by the professionals as a facilitator.

Other obstacles to delivering high quality care are the lack of the professionals’ knowledge and skills regarding actions for both prevention and management of PPH and team communication and collaboration [29]. Professionals often lack knowledge and skills about proactive actions to prevent exacerbation of PPH, but also about high risk factors for PPH. They sometimes overestimate their knowledge of the management of patients with PPH, but also their ability to estimate the blood loss without using a weighing-scale. It is known that estimating blood loss often means an underestimation [47]. Furthermore, insufficient team communication and collaboration, particularly the lack of clarity in leadership, were obstacles mentioned in all focus group interviews. Different studies reported the lack of effective leadership to promote and implement guideline recommendations as a barrier for effective guideline implementation [20, 48]. These obstacles could lead to inadequate team performance and a lack of standardised care, which is crucial in the emergency care setting, such as the management of PPH phase. Furthermore, the identified obstacles correspond with improvement factors identified in a simulated setting (unclear team roles, team communication problems, unidentified team leader, resulting in chaos and lack of documentation) [30]. However, whether it corresponds with the actual care is still unknown and has to be researched.

The difficulty of keeping up with literature due to lack of time is reported in this study and not only in the obstetrical field [21, 29]. Guidelines should facilitate the professional in this, but overall, professionals are often unaware of the existence or content of new guidelines [21, 29]. Prior education and team training to improve knowledge, skills, team communication and collaboration are important elements to improve PPH-care [49, 50]. Furthermore, since training on the total content of the guideline is often not feasible, training on the use of checklists and flowcharts could be more effective. All professionals mentioned both these factors as a facilitator in the delivery of high quality PPH-care.

The strong point of our study is its systematic approach to obtain information on influencing factors to the delivery of high quality care using both qualitative and quantitative research methods [39].^.^ Another strong point is the multidisciplinary approach, including all professionals involved in PPH-care, and the patients. We organised focus group discussions to identify potential obstacles to guideline adherence and performed an extensive questionnaire study among Dutch professionals involved in the PPH-care to quantify the prevalence and intensity of the different barriers. We realise that there are some limitations in our study as well. The international general applicability of our findings may be questionable. Nevertheless, our results apply to international guidelines, because we used guideline-based quality indicators, previously developed from international guidelines as a guide for the focus group interviews [51]. The limited response to the survey is another limitation; the quantitative results confirm the qualitative results, however. Therefore, it contributes to a broad support of the, yet to develop, tailor made strategy to improve the implementation of the national evidence-based PPH guideline and ATLS-based course instructions.

## Conclusion

In conclusion, obstacles as well as facilitators for the delivery of high quality PPH-care were identified, from both patient and professional perspective. Patient obstacles mainly concerned the lack of information provided by professionals. Checklists and flowcharts were mentioned as concrete tools to facilitate high quality care. For professionals, obstacles to the delivery of high quality PPH-care were identified in all domains, except the patient domain. These data can be used to develop a focused strategy to improve PPH-care. An additional step in the improvement strategy is to objectively measure the actual PPH-care.

## Abbreviations

PPH: Postpartum haemorrhage
ATLS: Advance-Trauma-Life-Support
OB-nurse: Obstetric nurse

## References

[1] Brace V, Kernaghan D, Penney G (2007). Learning from adverse clinical outcomes: major obstetric haemorrhage in Scotland, 2003–05. BJOG.

[2] Cristina Rossi A, Mullin P (2012). The etiology of maternal mortality in developed countries: a systematic review of literature. Arch Gynecol Obstet.

[3] Zwart JJ, Richters JM, Ory F, de Vries JI, Bloemenkamp KW, van Roosmalen J (2008). Severe maternal morbidity during pregnancy, delivery and puerperium in the Netherlands: a nationwide population-based study of 371,000 pregnancies. BJOG.

[4] Knight M, Callaghan WM, Berg C, Alexander S, Bouvier-Colle MH, Ford JB, Joseph KS, Lewis G, Liston RM, Roberts CL (2009). Trends in postpartum hemorrhage in high resource countries: a review and recommendations from the International Postpartum Hemorrhage Collaborative Group. BMC Pregnancy Childbirth.

[5] Lutomski JE, Byrne BM, Devane D, Greene RA (2012). Increasing trends in atonic postpartum haemorrhage in Ireland: an 11-year population-based cohort study. BJOG.

[6] The Dutch Perinatal Registry (PRN) [http://www.perinatreg.nl/home_english]

[7] Grol R, Grimshaw J (2003). From best evidence to best practice: effective implementation of change in patients’ care. Lancet.

[8] Deering S, Johnston LC, Colacchio K (2011). Multidisciplinary teamwork and communication training. Semin Perinatol.

[9] Buchan H, Sewell JR, Sweet M (2004). Translating evidence into practice. Med J Aust.

[10] Woolf SH, Grol R, Hutchinson A, Eccles M, Grimshaw J (1999). Clinical guidelines: potential benefits, limitations, and harms of clinical guidelines. BMJ.

[11] Penney G, Foy R (2007). Do clinical guidelines enhance safe practice in obstetrics and gynaecology?. Best Pract Res Clin Obstet Gynaecol.

[12] Grol R, Grol R (2013). Improving patient care : the implementation of change in health care.

[13] Berg CJ, Harper MA, Atkinson SM, Bell EA, Brown HL, Hage ML, Mitra AG, Moise KJ, Callaghan WM (2005). Preventability of pregnancy-related deaths: results of a state-wide review. Obstet Gynecol.

[14] Cantwell R, Clutton-Brock T, Cooper G, Dawson A, Drife J, Garrod D, Harper A, Hulbert D, Lucas S, McClure J (2011). Saving Mothers’ Lives: Reviewing maternal deaths to make motherhood safer: 2006–2008. The Eighth Report of the Confidential Enquiries into Maternal Deaths in the United Kingdom. BJOG.

[15] Johanson R, Cox C, Grady K, Howell C (2009). Managing Obstetric Emergency and Trauma; The MOET Course Manual.

[16] Clark SL (2012). Strategies for reducing maternal mortality. Semin Perinatol.

[17] Chaillet N, Dube E, Dugas M, Audibert F, Tourigny C, Fraser WD, Dumont A (2006). Evidence-based strategies for implementing guidelines in obstetrics: a systematic review. Obstet Gynecol.

[18] Wensing M, van der Weijden T, Grol R (1998). Implementing guidelines and innovations in general practice: which interventions are effective?. Br J Gen Pract.

[19] Dupont C, Deneux-Tharaux C, Touzet S, Colin C, Bouvier-Colle MH, Lansac J, Thevenet S, Boberie-Moyrand C, Piccin G, Fernandez MP (2011). Clinical audit: a useful tool for reducing severe postpartum haemorrhages?. Int J Qual Health Care.

[20] Grol R, Wensing M (2004). What drives change? Barriers to and incentives for achieving evidence-based practice. Med J Aust.

[21] Cabana MD, Rand CS, Powe NR, Wu AW, Wilson MH, Abboud PA, Rubin HR (1999). Why don’t physicians follow clinical practice guidelines? A framework for improvement. JAMA.

[22] Stienen JJ, Ottevanger PB, Wennekes L, van de Schans SA, Dekker HM, Blijlevens NM, van der Maazen RW, van Krieken JH, Hermens RP (2014). Delivering high-quality care to patients with a non-Hodgkin’s lymphoma: barriers perceived by patients and physicians. Neth J Med.

[23] van Peperstraten AM, Nelen WL, Hermens RP, Jansen L, Scheenjes E, Braat DD, Grol RP, Kremer JA (2008). Why don’t we perform elective single embryo transfer? A qualitative study among IVF patients and professionals. Hum Reprod.

[24] van den Boogaard NM, van den Boogaard E, Bokslag A, van Zwieten MC, Hompes PG, Bhattacharya S, Nelen W, van der Veen F, Mol BW (2011). Patients’ and professionals’ barriers and facilitators of tailored expectant management in subfertile couples with a good prognosis of a natural conception. Hum Reprod.

[25] van Peperstraten AM, Hermens RP, Nelen WL, Stalmeier PF, Scheffer GJ, Grol RP, Kremer JA (2008). Perceived barriers to elective single embryo transfer among IVF professionals: a national survey. Hum Reprod.

[26] Wensing M, Laurant M, Hulscher M, Grol R (1999). Theory an Practice of Clinical Guidelines of Implementation; Chapter 6: Methods for identifying barriers and facilitators for implementation. Danish Institute for Heath Services Research and Development.

[27] Woiski MD, Scheepers HJ, Liefers J, Lance M, Middeldorp JM, Lotgering FK, Grol RP, Hermens RP (2015). Guideline-based development of quality indicators for prevention and management of postpartum hemorrhage. Acta Obstet Gynecol Scand.

[28] Toussaint ND, Pedagogos E, Beavis J, Becker GJ, Polkinghorne KR, Kerr PG (2011). Improving CKD-MBD management in haemodialysis patients: barrier analysis for implementing better practice. Nephrol Dial Transplant.

[29] Belizan M, Meier A, Althabe F, Codazzi A, Colomar M, Buekens P, Belizan J, Walsh J, Campbell MK (2007). Facilitators and barriers to adoption of evidence-based perinatal care in Latin American hospitals: a qualitative study. Health Educ Res.

[30] Faulkner B (2013). Applying lean management principles to the creation of a postpartum hemorrhage care bundle. Nurs Womens Health.

[31] Britten N, Shaw A (1994). Patients’ experiences of emergency admission: how relevant is the British government’s Patients Charter?. J Adv Nurs.

[32] Harrison MJ, Kushner KE, Benzies K, Rempel G, Kimak C (2003). Women’s satisfaction with their involvement in health care decisions during a high-risk pregnancy. Birth.

[33] Greenfield S, Kaplan S, Ware JE (1985). Expanding patient involvement in care. Effects on patient outcomes. Ann Intern Med.

[34] Renders CM, Valk GD, Griffin SJ, Wagner EH, Van Eijk JT, Assendelft WJ (2001). Interventions to improve the management of diabetes in primary care, outpatient, and community settings: a systematic review. Diabetes Care.

[35] Leblanc JM, Kane-Gill SL, Pohlman AS, Herr DL (2012). Multiprofessional survey of protocol use in the intensive care unit. J Crit Care.

[36] Hussain T, Michel G, Shiffman RN (2009). The Yale Guideline Recommendation Corpus: a representative sample of the knowledge content of guidelines. Int J Med Inform.

[37] Codish S, Shiffman RN: A model of ambiguity and vagueness in clinical practice guideline recommendations. AMIA Annu Symp Proc. 2005:146–150. PMC1560665 16779019

[38] Deneux-Tharaux C, Dupont C, Colin C, Rabilloud M, Touzet S, Lansac J, Harvey T, Tessier V, Chauleur C, Pennehouat G (2010). Multifaceted intervention to decrease the rate of severe postpartum haemorrhage: the PITHAGORE6 cluster-randomised controlled trial. BJOG.

[39] Audureau E, Deneux-Tharaux C, Lefevre P, Brucato S, Morello R, Dreyfus M, Bouvier-Colle MH (2009). Practices for prevention, diagnosis and management of postpartum haemorrhage: impact of a regional multifaceted intervention. BJOG.

[40] Cameron CA, Roberts CL, Bell J, Fischer W (2007). Getting an evidence-based post-partum haemorrhage policy into practice. Aust N Z J Obstet Gynaecol.

[41] Fleuren M, van der Meulen M, Grol R, de Haan M, Wijkel D (1998). Does the care given by general practitioners and midwives to patients with (imminent) miscarriage meet the wishes and expectations of the patients?. Int J Qual Health Care.

[42] Sinuff T, Eva KW, Meade M, Dodek P, Heyland D, Cook D (2007). Clinical practice guidelines in the intensive care unit: a survey of Canadian clinicians’ attitudes. Can J Anaesth.

[43] Graham ID, Logan J, Davies B, Nimrod C (2004). Changing the use of electronic fetal monitoring and labor support: a case study of barriers and facilitators. Birth.

[44] Fausett MB, Propst A, Van Doren K, Clark BT (2011). How to develop an effective obstetric checklist. Am J Obstet Gynecol.

[45] Norton EK, Rangel SJ (2010). Implementing a pediatric surgical safety checklist in the OR and beyond. AORN J.

[46] Savel RH, Goldstein EB, Gropper MA (2009). Critical care checklists, the Keystone Project, and the Office for Human Research Protections: a case for streamlining the approval process in quality-improvement research. Crit Care Med.

[47] Schorn MN (2010). Measurement of blood loss: review of the literature. J. Midwifery Womens Health.

[48] Sinuff T, Cook D, Giacomini M, Heyland D, Dodek P (2007). Facilitating clinician adherence to guidelines in the intensive care unit: A multicenter, qualitative study. Crit Care Med.

[49] Siassakos D, Draycott T, Montague I, Harris M (2009). Content analysis of team communication in an obstetric emergency scenario. J. Obstet. Gynecol.

[50] Osman H, Campbell OM, Nassar AH (2009). Using emergency obstetric drills in maternity units as a performance improvement tool. Birth.

[51] Sheldon WR, Blum J, Vogel JP, Souza JP, Gulmezoglu AM, Winikoff B, WHO Multicountry Survey on Maternal and Newborn Health Research Network (2014). Postpartum haemorrhage management, risks, and maternal outcomes: findings from the World Health Organization Multicountry Survey on Maternal and Newborn Health. BJOG-Int J Obstet Gy.

